# Long-Term Antibody Response to SARS-CoV-2 in Children

**DOI:** 10.1007/s10875-022-01355-w

**Published:** 2022-09-19

**Authors:** Gabor A. Dunay, Madalena Barroso, Mathias Woidy, Marta K. Danecka, Geraldine Engels, Katharina Hermann, Friederike S. Neumann, Kevin Paul, Jan Beime, Gabriele Escherich, Kristin Fehse, Lev Grinstein, Franziska Haniel, Luka J. Haupt, Laura Hecher, Torben Kehl, Christoph Kemen, Markus J. Kemper, Robin Kobbe, Aloisa Kohl, Thomas Klokow, Dominik Nörz, Jakob Olfe, Friderike Schlenker, Jessica Schmiesing, Johanna Schrum, Freya Sibbertsen, Philippe Stock, Stephan Tiede, Eik Vettorazzi, Dimitra E. Zazara, Antonia Zapf, Marc Lütgehetmann, Jun Oh, Thomas S. Mir, Ania C. Muntau, Søren W. Gersting, the C19.CHILD Study Group, Stefanie Etzold, Ingrid Goebel, Armin Günther, Pia-Sophie Kantor, Lea Bandel, Markus Baumanns, Pia Dähler, Barbara Dwenger, Annika Erdmann, Sophia Hegselmann, Kai Hornig, Johanna Jipp, Pia Kirkerup, Michael Krumm, Pelin Kurnaz, Sabine Pasterkamp, Anna Serve, Daniel Tegtmeyer, Julia Terstegen, Ceri Theresa Wiedling, Madelaine Wingerath, Stefan Rutkowski, Beate Winkler, Florian Arndt, Stefan Blankenberg, Daniel Diaz, Peer Hauck, Rainer G. Kozlik-Feldmann, Götz C. Müller, Veronika C. Stark, Peter Wiegand, Martin Aepfelbacher, Kathrin Cermann, Armin Hoffmann, Johannes K.-M. Knobloch, Marylyn M. Addo, Petra Arck, Anke Diemert, Corinna Cramer, Kurt Hecher, Peter Höger, Anja Große Lordemann, Maria-Dorothee Neumann, Bernd Hartz, Anne Kruchen, Ingo Müller, Michael Boettcher, Konrad Reinshagen, Carolin Stiel, Katharina Wenke, Joana Adler Fernandes de Abreu, Marlies Bergers, Martin Blohm, Philipp Deindl, Theresa Harbauer, Cornelius Rau, Dominique Singer, Marianne Klokow, Julia Strauss, Stefan Blankenberg, Ines Schäfer, Jens Vogel, Maximilian Noelle-Wying, Julian Schulze zur Wiesch, Davor Lessel, Caroline Schmitt, Christian Drosten

**Affiliations:** 1 University Children’s Research, UCR@Kinder-UKE, University Medical Center Hamburg-Eppendorf, Martinistr. 52, 20251 Hamburg, Germany; 2 Department of Pediatrics, Kinder-UKE, University Medical Center Hamburg-Eppendorf, Martinistr. 52, 20251 Hamburg, Germany; 3 Department of Pediatric Hematology and Oncology, University Medical Center Hamburg-Eppendorf, Martinistr. 52, 20251 Hamburg, Germany; 4 Department of Pediatric Cardiology, University Medical Center Hamburg-Eppendorf, Martinistr. 52, 20251 Hamburg, Germany; 5 Wilhelmstift Children’s Hospital, Liliencronstraße 130, 22149 Hamburg, Germany; 6 Asklepios Klinik Nord – Heidberg, Tangstedter Landstraße 400, 22417 Hamburg, Germany; 7 Institute for Infection Research and Vaccine Development (IIRVD), University Medical Center Hamburg-Eppendorf, Martinistr. 52, 20251 Hamburg, Germany; 8 Institute of Medical Microbiology, Virology and Hygiene, University Medical Center Hamburg-Eppendorf, Martinistr. 52, 20251 Hamburg, Germany; 9 Altona Children’s Hospital, Bleickenallee 38, 22763 Hamburg, Germany; 10 Institute of Medical Biometry and Epidemiology, University Medical Center Hamburg-Eppendorf, Martinistr. 52, 20251 Hamburg, Germany; 11 Department of Obstetrics and Prenatal Medicine, Division for Experimental Feto-Maternal Medicine, University Medical Center Hamburg-Eppendorf, Martinistr. 52, 20251 Hamburg, Germany

**Keywords:** Pediatric, Longitudinal, Serology, Ancestral, COVID-19

## Abstract

Almost 2 years into the pandemic and with vaccination of children significantly lagging behind adults, long-term pediatric humoral immune responses to SARS-CoV-2 are understudied. The C19.CHILD Hamburg (COVID-19 Child Health Investigation of Latent Disease) Study is a prospective cohort study designed to identify and follow up children and their household contacts infected in the early 2020 first wave of SARS-CoV-2. We screened 6113 children < 18 years by nasopharyngeal swab-PCR in a low-incidence setting after general lockdown, from May 11 to June 30, 2020. A total of 4657 participants underwent antibody testing. Positive tests were followed up by repeated PCR and serological testing of all household contacts over 6 months. In total, the study identified 67 seropositive children (1.44%); the median time after infection at first presentation was 83 days post-symptom onset (PSO). Follow-up of household contacts showed less than 100% seroprevalence in most families, with higher seroprevalence in families with adult index cases compared to pediatric index cases (OR 1.79, *P* = 0.047). Most importantly, children showed sustained seroconversion up to 9 months PSO, and serum antibody concentrations persistently surpassed adult levels (ratio serum IgG spike children vs. adults 90 days PSO 1.75, *P* < 0.001; 180 days 1.38, *P* = 0.01; 270 days 1.54, *P* = 0.001). In a low-incidence setting, SARS-CoV-2 infection and humoral immune response present distinct patterns in children including higher antibody levels, and lower seroprevalence in families with pediatric index cases. Children show long-term SARS-CoV-2 antibody responses. These findings are relevant to novel variants with increased disease burden in children, as well as for the planning of age-appropriate vaccination strategies.

**Supplementary Information:**

The online version contains supplementary material available at 10.1007/s10875-022-01355-w.

## Introduction

The severe acute respiratory syndrome coronavirus 2 (SARS-CoV-2) pandemic has been responsible for over five million deaths [1]. Severe disease due to acute coronavirus disease 2019 (COVID-19) is rare in children and teenagers [2–4], and the pediatric inflammatory multisystem syndrome (PIMS, also termed MIS-C) attributable to SARS-CoV-2 only occurs in a small minority of cases [5, 6]. Vaccination of adults and teenagers is now widespread even though vaccination rates show large regional differences [1]. Vaccination of younger children above the age of five years [7] has been ongoing for several months (the USA) to several weeks (Europe) and even longer in China at the time of this report. Emerging viral variants, especially the most recent omicron variant (B.1.1.52), appear to escape neutralizing antibodies provided by vaccination [8]. Furthermore, the pediatric population may be at increased risk, because initial reports of the omicron variant suggest an increased hospitalization rate in children compared to earlier variants [9].

Despite the pressing need to better understand long-term antibody responses in children, longitudinal studies over longer periods after natural infection are almost completely lacking in this age group. Here, we present a prospective cohort study of 67 convalescent children and their household contacts including a quantitative follow-up of antibody responses in household clusters up to 9 months after symptomatic infection. The children were recruited in the C19.CHILD Hamburg Study (COVID-19 Child Health Investigation of Latent Disease), in which over six thousand children under eighteen years of age were screened for acute SARS-CoV-2 infection by PCR and 4657 serological tests were performed. The recruitment to the study followed a general lockdown after the initial wave of ancestral SARS-CoV-2 infection in 2020.

## Methods

### Study Population and Ethical Approval

We conducted an observational prospective multi-center cohort study of children under eighteen years of age in all five pediatric hospitals of the Free and Hanseatic City of Hamburg, Germany: AKK Altonaer Kinderkrankenhaus, Helios Mariahilf Kinderklinik, Klinik für Kinder- und Jugendmedizin der Asklepios Klinik Nord-Heidberg, Katholisches Kinderkrankenhaus Wilhelmstift, and Werner und Michael Otto Universitätskinderklinik at the University Medical Center Hamburg-Eppendorf (Kinder-UKE) from May 11 to June 30, 2020. As part of a general lockdown, school and day care facilities in Hamburg were closed from March 1 to at least May 18, 2020, so that recruitment to the study began shortly before lifting these measures. Patients presenting to the study hospitals were invited to participate. In addition, children from the community were welcomed to enroll through the C19.CHILD Study Clinic at Kinder-UKE. The study population consisted of (i) in- and outpatients aged 0 to < 18 years of all pediatric hospitals in Hamburg, Germany; (ii) children aged 0 to < 18 years as volunteers; (iii) participants of other studies (Prenatal Investigation of Children’s Health-PRINCE study, Hamburg City Health Study) aged 0 to < 18 years; and (iv) children, who were household contacts of SARS-CoV-2 positive study participants.

For recruitment of volunteers, appeals were launched via social media and consent forms and information sheets were distributed via the websites c19child.hamburg and c19child.de. In addition, information material was distributed to schools, kindergartens, and public institutions in the Hamburg area. Filling out a standardized questionnaire, as well as taking a nasopharyngeal swab for the PCR detection of SARS-CoV-2 RNA, was obligatory for all participants during the screening phase, whereas a venous blood draw for the detection of antibodies directed against SARS-CoV-2 was optional.

Recruitment and consent procedures were equivalent in all participating hospitals. A written informed consent was obtained for each participating subject. A written informed consent was obtained from parents or guardians in all cases, from children over 7 years whenever possible; for children, also consent in spoken word was accepted. The inclusion criteria were as follows: (i) children or teenagers aged 0–18 years; (ii) patient in one of the participating centers or volunteer in the central C19.CHILD Study Clinic; (iii) informed consent from parents or guardians; (iv) informed consent from children > 7 years (unless not capable). The exclusion criteria were as follows: (i) prematurity < 37 weeks of gestation; (ii) informed consent of parents or guardians not possible in spoken word or otherwise; (iii) informed consent not given. The study was approved by the local ethical committee of Hamburg (reference number: PV7336) and published at clinicaltrials.gov (NCT04534608).

Children with a positive PCR and/or antibody test, as well as their household contacts (referred to as family members), were invited to three follow-up appointments. The follow-ups were carried out at the earliest possible timepoint within days of screening, then at three and six months after screening at the C19.CHILD Study Clinic. Here, an extended questionnaire including a detailed history of infection was completed, and nasopharyngeal swabs and venous blood samples were collected from all family members willing to participate for SARS-CoV-2 viral RNA and antibody testing.

### Sample Processing and Viral RNA Detection

Viral RNA was extracted from nasopharyngeal swabs using a Tecan Freedom Evo liquid handler system (Tecan) and the NucleoSpin 96 Virus Core Kit (Macherey–Nagel; refer to *Supplementary Methods* for details). For the detection of the SARS-CoV-2 RNA, the RealStar SARS-CoV-2 RT-PCR Kit 1.0 (Altona Diagnostics) was used according to the manufacturer’s recommendations. The dual target assay detects the viral E-gene (B-βCoV-specific) and S-gene (SARS-CoV-2-specific). Samples with positivity in both gene targets were considered positive. SARS-CoV-2 RNA detection of the follow-up participants was performed using the ECDC recommended E-gene assay [10] adapted for high-throughput PCR (Cobas 6800, Roche), as described by S. Pfefferle et al. [11].

### Antibody Measurements

SARS-CoV-2-specific antibodies (IgA/IgM/IgG) against the viral nucleocapsid protein were detected in sample serum using the Elecsys® Anti-SARS-CoV-2 Ig assay (Roche) on the cobas e411 system (Roche). Positive results were confirmed in the same serum sample with the LIAISON® SARS-CoV-2 IgG serology test (DiaSorin). This alternative assay allows for quantification of SARS-CoV-2 antibodies (IgG) against the S1 and S2 subunits of the spike protein. Assays were performed IVD conform according to the manufacturer’s recommendations including thresholds for positivity (see *Supplementary Methods*). Following the guidelines of the CDC for a low prevalence setting (< 5%) [12], an orthogonal testing algorithm was used; therefore, samples were only considered seropositive if positive results were obtained with both assays. Antibody testing was performed identically with both tests at all follow-up appointments for each participating family member.

### Statistical Analysis

Frequency of demographic and clinical features was reported for study subjects stratified according to SARS-CoV-2 test results or age groups. Age is shown as mean ± standard deviation, and categorical variables as counts and/or percentages. The primary aim was to estimate the prevalence of SARS-CoV-2 in children and the sample size was justified in this respect. Since only one child was acutely infected, this primary question cannot be answered. Therefore, all analyses are secondary and explorative. Accordingly, the *P*-values are not adjusted for multiplicity and are only reported as descriptive measures. Children from the same family were included in the study. This creates a cluster structure that must be adequately considered in the statistical methodology. We therefore used mixed logistic models with the family as random intercept, where appropriate. Another issue is the low number of events, which can lead to separation problems and thus instable estimates. We addressed this by using penalized models provided in the R package GLMMadaptive. For the longitudinal analysis of serum antibody concentrations, a generalized linear mixed model was used with random effects for individuals nested within families. The tweedie distribution, which is implemented in the R package glmmTMB, was chosen in this model to account for measurements below the detection limit and, additionally, a zero-inflation was allowed to reflect negative test results. The results were robust with respect to the link function, which was verified in sensitivity analyses. Analyses were performed using R software (version 4.0.2) and GraphPad Prism (version 8.4).

## Results

We screened 6113 children under 18 years of age in the Free and Hanseatic City of Hamburg, Germany (population 1,845,017 on April 30, 2020) within a defined time period (May 11–June 30, 2020) and included 5908 study participants from 4506 families in the C19.CHILD study. Children were recruited regardless of the presence or absence of any COVID-19 symptoms. The study included 3145 (53%) volunteers recruited through the C19.CHILD Study Clinic at Kinder-UKE (of these 345 participants in other studies) and 2763 (47%) subjects who presented at one of the Hamburg pediatric hospitals for reasons independent of this study (also see Supplementary Methods). In case of a positive PCR and/or antibody test, children were invited for follow-up tests along with all contact persons living in the same household.

One child tested PCR positive for SARS-CoV-2. Two antibody tests were used for this study. For analysis, the Roche nucleocapsid protein total IgA/IgM/IgG test was defined as screening antibody test (available for 4657 participants, 79% of total included) and the DiaSorin S1/S2 spike IgG was defined as confirmatory test. A total of 67 children, 1.44% of all tested, had a positive antibody reaction in both antibody tests in the screening. Participants were recalled for a second testing shortly after positive antibody screening, typically within five days (mean 5.31). Recall testing of seropositives in the screening phase was available for 48 children; all tests remained positive as determined by both methods (Supplementary Table S2) and one child who was only positive in one of the two antibody tests during screening (Diasorin) tested seropositive (Diasorin and Roche positive) in the follow-up. Seven children refused repeat blood draws at immediate follow-up; hence, results from the screening antibody test were used for further analyses (Supplementary Table S2).

In this cohort, the seroprevalence of SARS-CoV-2 increased with age; the mean age of seropositive children was 2 years higher than that of seronegative children (10.3 vs. 8.3 years, *P* = 0.001, Table 1). We observed a continuous age effect, where seroprevalence increased with an odds ratio of 1.11 per year of age (95% CI 1.03–1.21, *P* = 0.009 Supplementary Figure 1) in the cohort of screened children under 18 years. Inclusion of more than one child per family created a cluster structure, which we considered by applying mixed logistic regression models.

**Table 1:** Baseline characteristics of the study population. Age is given as mean (standard deviation); all other are counts (percent) or counts/*N* (percent) in case of missing data. Age is compared using Student’s *t*-test; all binary data is compared using Fisher’s exact test

	Negative in either test (*N* = 4590)	Positive Roche & DiaSorin (*N* = 67)	Total (*N* = 4657)	*p*-value
Age (years)	8.3 (5.0)	10.3 (4.4)	8.3 (5.0)	0.001
Female gender	2172 (47.3%)	33 (49.3%)	2205 (47.3%)	0.806
Underlying medical conditions	1334 (29.1%)	16 (23.9%)	1350 (29.0%)	0.416
Age-appropriate vaccination complete	4088/4428 (92.3%)	65/66 (98.5%)	4153/4494 (92.4%)	0.060
Contact to persons with known SARS-CoV-2 Infection	92/1712 (5.4%)	27/33 (81.8%)	119/1745 (6.8%)	< 0.001

Seroprevalence did not show any gender differences in this cohort (Table 1). Children and adolescents recruited in the participating hospitals often suffered from a complex chronic disease (29% indicated an underlying medical condition, detailed in Supplementary Table S1). One might expect a high proportion of vulnerable individuals in the C19.CHILD study population regarding infection rates or severity of disease [13]. However, children with an underlying medical condition representing almost one-third of the study cohort did not show a difference in the seroprevalence (Table 1, Supplementary Table S1). When comparing self-reported, age-appropriate vaccinations with seroprevalence, children with a complete vaccination status were slightly more likely to be seropositive than children with missed vaccinations (Table 1, 98.5 vs. 92.3%, *P* = 0.060). Participants and their guardians were asked to report on any persistent symptoms associated with COVID-19 in the last 14 days before screening. Notably, the only symptom that was more common in seropositive children was loss of taste (seronegatives 15/4590, 1.3%, seropositives 3/67, 21.4%, *P* = 0.001).

We further looked at the effect of self-reported exposure to persons with confirmed SARS-CoV-2 infection on seroprevalence. Exposure was reported for 119 children from whom antibody tests were also available. Twenty-seven of these children had a confirmed positive screening antibody test (22.7%) and 20 were available for recall testing, all of whom tested positive for both nucleocapsid IgA/IgG/IgM and spike IgG. There was no statistically significant difference of seroprevalence for children aged 12– < 18 years (seroprevalence 48.1%, OR 3.2, 95% CI 0.5–18.9) as well children aged 6– < 12 years (seroprevalence 40.7%, OR 1.1, 95% CI 0.2–5.6) compared to children aged 0– < 6 years (seroprevalence 11.1%, OR 1) after contact to SARS-CoV-2. Furthermore, seroprevalence was higher if the contact had been within the family (92.6% of seropositives after contact, OR 20.9, 95%, CI 3.0–143.8), than if contacts had been outside the family (7.4% of seropositives after contact, OR 1, Table 2).

**Table 2:** Impact of age and type of contact to a person infected with SARS-CoV-2 on seroprevalence. Odds ratios are estimates from a multiple mixed logistic model

	Negative in either test (*N* = 92)	Positive Roche & DiaSorin (*N* = 27)	Total (*N* = 119)	Odds ratio (95% CI)
Age (grouped)				
0 to < 6	21 (22.8%)	3 (11.1%)	24 (20.2%)	1 (reference)
6 to < 12	42 (45.7%)	11 (40.7%)	53 (44.5%)	1.1 (0.2, 5.6)
12 to < 18	29 (31.5%)	13 (48.1%)	42 (35.3%)	3.2 (0.5, 18.9)
Type of contact				
Outside family	60 (65.2%)	2 (7.4%)	62 (52.1%)	1 (reference)
Inside family	32 (34.8%)	25 (92.6%)	57 (47.9%)	20.9 (3.0, 143.8)

**Fig. 1:**
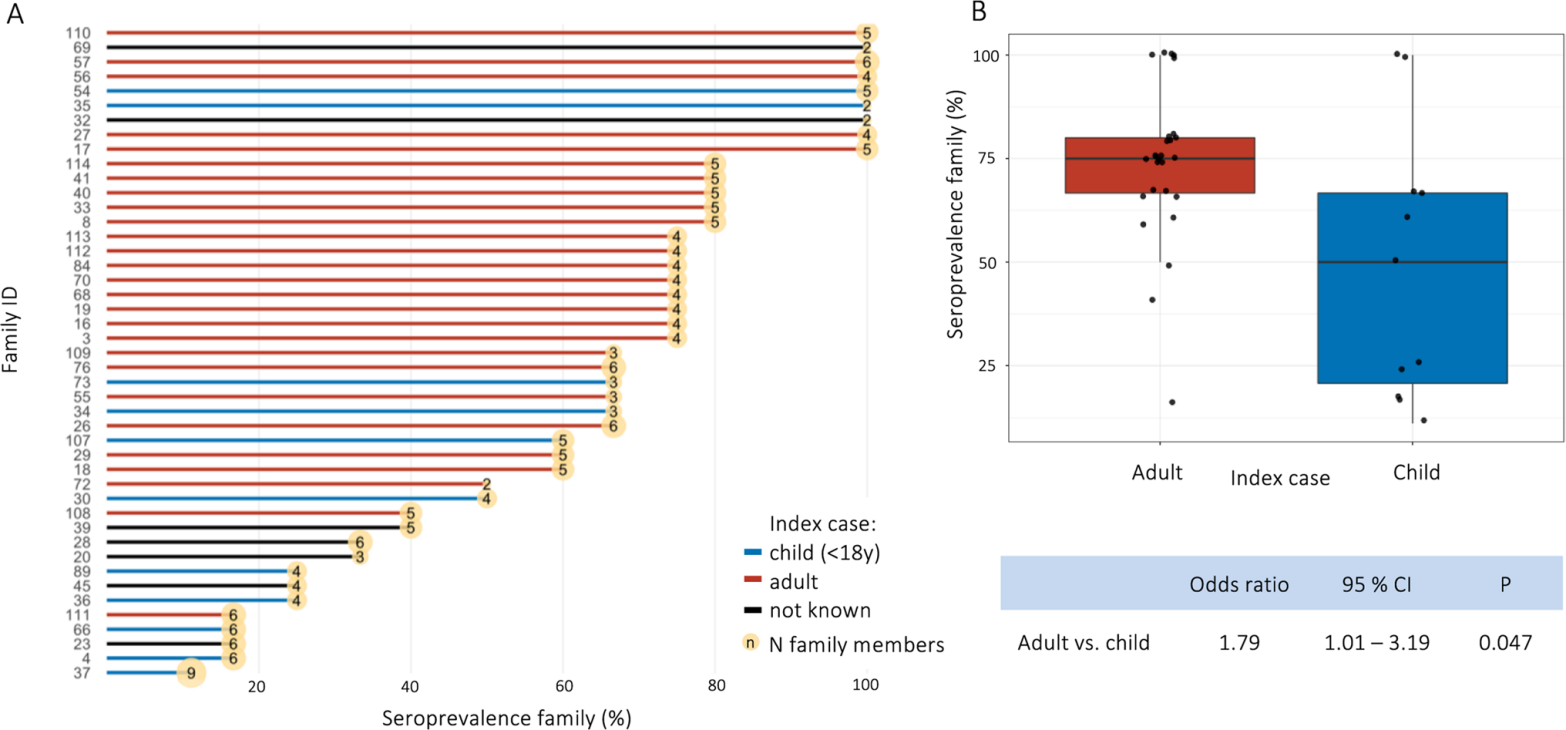
Seroprevalence among household contacts. **A** Seroprevalence within individual families recalled for follow-up. The seroprevalence, calculated by the seropositive household contacts divided by all household contacts, is shown on the *x*-axis. Families are depicted by their respective ID and arranged by seroprevalence from top to bottom. At the end of each bar, the number of family members participating in the study is shown. Families are colored according to the age group of the respective index case (child < 18 years or adult > 18 years), where applicable. Seven families were not able to recall the index case. For three families (ID: 28, 29, 73), at least one family member refused blood draw. **B** Seroprevalence within families by age group of the index case. Families with an adult index case had a higher seroprevalence compared to families with a pediatric index case (mean seroprevalence 73.8 vs. 48.9%). To model seroprevalence with the type of index case, a logistic regression model was applied. Results are shown in the lower panel (odds ratio 1.79, 95% CI 1.01–3.19, *P* = 0.047)

Next, seroprevalence among household contacts was analyzed. We recalled 45 families for an initial follow-up visit within days after screening (median 83 days post-symptom onset, range 21–121 days based on available data from 39 families). Seroprevalence within single families was 100% only in a minority of cases (Fig. 1A, Supplementary Table S2), where seropositivity was again defined based on a positive result for both nucleocapsid IgA/IgG/IgM and spike IgG. Thirty-eight families were able to identify the first infected person in the household (index case) based on the beginning of symptoms or a known contact to an infected person. Notably, when comparing seroprevalence between individual families, we found that they were lower when the index case was younger than 18 years than for adult index cases (mean seroprevalence 48.9 vs. 73.8%, Fig. 1B). Applying logistic regression to model seroprevalence with the type of index case as predictor confirmed these findings (OR 1.79, 95% CI 1.01–3.19, *P* = 0.047).

The follow-up phase of this study included further recall appointments for the 45 recall families, at 3 months and 6 months after screening (median 173 days, range 112–243 days, and median 254 days, range 193–279 days, post-symptom onset, PSO). Here, PCR and serologic tests were repeated for family members. Twelve families have been lost to follow-up by the end of the study, and in some cases, not all family members were present at every appointment. Six families were excluded from analysis on account of no data available on the date of symptom onset. Despite a steep rise in incidence in Hamburg, Germany, in November–December 2020, no acute infection could be detected in any of the study participants by PCR during follow up.

Longitudinal analysis of antibodies showed that almost all children and adults who were seropositive at the time of the first follow-up retained detectable SARS-CoV-2 antibodies at the time of the third follow up 6 months later (> 193 days after symptom onset). This was observed for both types of antibodies measured in this study, with nucleocapsid IgA/IgG/IgM seropositivity retained in 100% of adults (*n* = 36), and 97.6% of children (*n* = 41) and spike IgG seropositivity retained in 94.4% of adults (*n* = 36), and 100% of children (*n* = 41) at six months after screening in those initially positive in both tests.

**Fig. 2:**
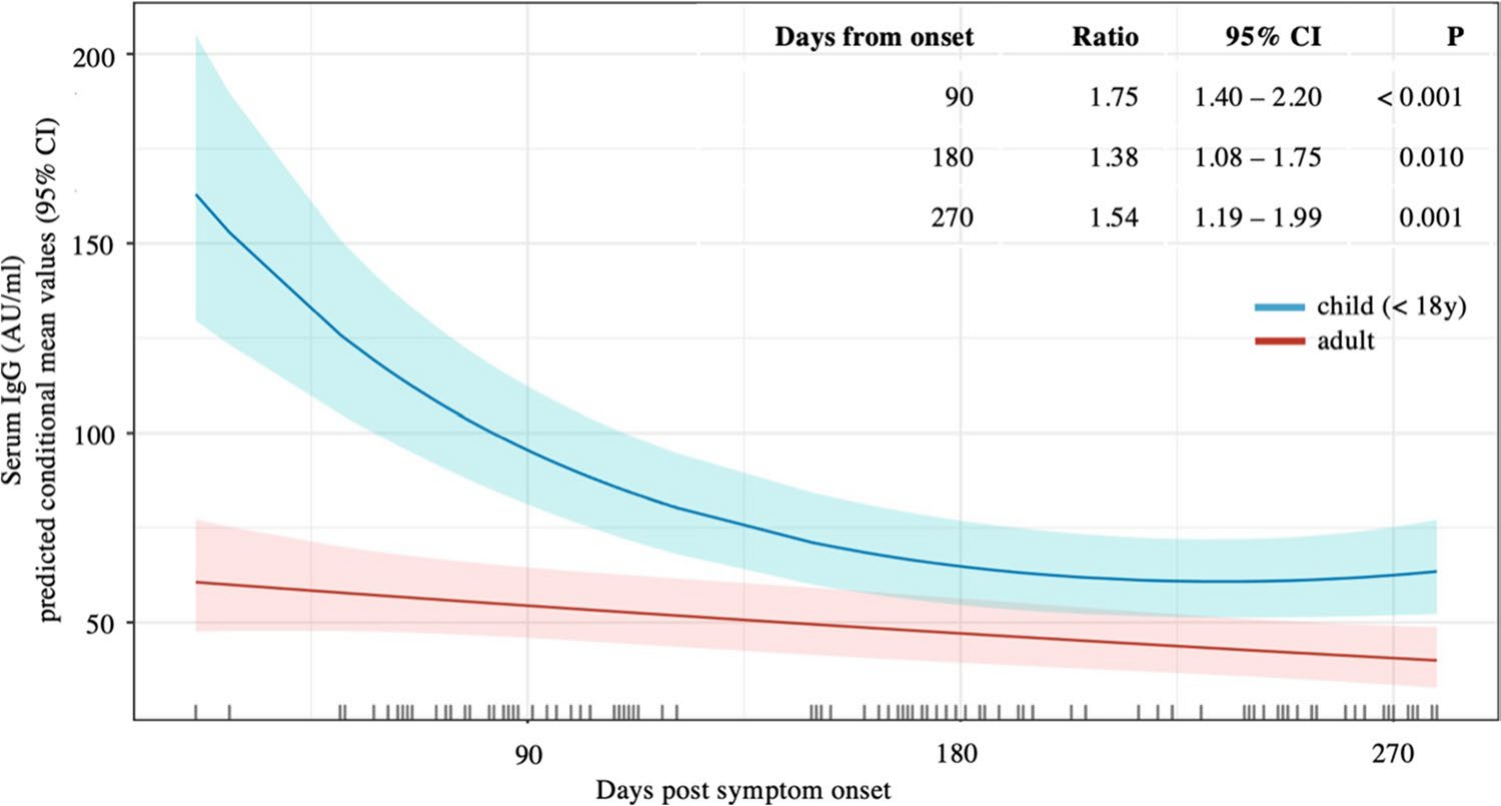
Longitudinal follow-up of serum antibody concentrations of SARS-CoV-2 anti-spike IgG in adults and children. Observation times post-symptom onset are indicated on the *x*-axis and are calculated per family. The *y*-axis is showing predicted conditional mean values (bold curve) of the serum SARS-CoV-2 anti-spike IgG concentration as determined by the zero-inflated tweedie mixed model. The shaded areas flanking the curves indicate 95% confidence intervals. Predicted conditional means are dependent on zero-inflation. Table in the top right section showing estimated mean ratio of predicted conditional mean values children vs. adults at 90, 180, and 270 days post-symptom onset, along with 95% confidence intervals and corresponding *P* values. Color coding for adults and children is indicated

The spike IgG test allowed for quantitative assessment of serum antibody concentrations. To follow changes in antibody levels of anti-spike IgG over time, statistical modeling using the zero-inflated tweedie-mixed model was applied (Fig. 2). Time points at 90 days, 180 days, and 270 days PSO were chosen for comparison between adults and children, matching the observed time frame in the study cohort (Fig. 2). At all of these time points, children had a higher antibody concentration than adult family members (ratio children vs. adults 90 days PSO 1.75, 95% CI 1.40–2.20, *P* < 0.001; 180 days PSO 1.38, 95% CI 1.08–1.75, *P* = 0.01; 270 days PSO 1.54, 95% CI 1.19–1.99, *P* = 0.001). During the observed time period, both children and adults had decreasing antibody concentrations over time. For children, the decrease was faster and more pronounced initially (Fig. 2).

## Discussion

Here, we report the results of the C19.CHILD study, a large prospective seroprevalence study for SARS-CoV-2 targeting children in a low-incidence setting. Previous seroprevalence studies carried out under lockdown have compared seroprevalence between children and adults, and found these to be lower in children [14–16]. These findings must be interpreted with caution because different contact patterns of children under lockdown may play a substantial role. A large, population-based seroepidemiological study out of Wuhan, China, a place of high initial incidence, described similar seroprevalence in children and older age groups [17]. In our cohort, seroprevalence among children increased with age, as had been the case for other cohorts of the ancestral SARS-CoV-2, including a seroprevalence study in a similar time interval from the south of Germany [14, 15, 18–20]. These findings may have been specific to the ancestral variant of SARS-CoV-2 and the circumstances governing the early 2020 first wave of coronavirus infections including few imported cases followed by lockdown. Subsequent infection waves, including novel viral variants, showed an increase in seropositivity in children [21], probably based on the reopening of schools and daycare. At the same time, comparison of seroprevalence in children to adults and older teenagers became less meaningful with the spread of vaccination [21].

We have found that in the case of exposure to a confirmed case of SARS-CoV-2 infection, younger children (zero to under six years) had a lower seroprevalence than older children, although the results did not reach statistical significance. Consistently, lower secondary attack rates have been reported in children than in adults [22–24]. A large study on SARS-CoV-2 transmission dynamics in two Indian states, which included children, provided evidence that contacts between people of similar age are more likely to lead to infection [25]. Thus, a lack of contact between children and their similar-age peers in a lockdown scenario might explain the differences between our data and studies in similar low-incidence settings [15, 16] compared to seroprevalence studies in places with high initial incidence [17].

We report that seroprevalence within individual families with index persons below the age of eighteen years were considerably lower. In our cohort, we made use of the relatively high availability of data on the index person in each family, as in the early phase of the pandemic the source of infection could still be identified in most cases. Most contact tracing and household cluster studies of SARS-CoV-2 have suggested a lower risk of transmission by children [25–30]. In a large population-based cohort study spanning the whole of 2020, older children had a lower risk of transmitting SARS-CoV-2 than younger children, but a comparison with adults was not described [31]. Again, these differences may relate to the highly different contact patterns of children under a general lockdown. In Hamburg, Germany, general school and day care closure lasted from March 1 to at least May 18, 2020, and recruitment to the C19.CHILD Study began on May 11, shortly before lockdown was lifted. In this period, contacts for children outside their own families were very limited and incidence of novel SARS-CoV-2 infections was low (Supplementary Figure S2). Infection of most index persons in the C19.CHILD cohort probably happened before the lockdown, which is supported by patient histories regarding symptomatic illness in late February and early March (Supplementary Table S2). Subsequently, spread of infection would mostly have been limited to family clusters. As awareness of COVID-19 was high in the public, it may be assumed that symptomatic children, especially teenagers, would have been isolated at home (to the extent possible). The isolation of adult caregivers, however, may have been more difficult. As a result, less transmission would have occurred from children than adults. Alternatively, a less efficient biological transmission of the virus by children in the lockdown setting could explain our findings. As viral loads in nasopharyngeal swabs appear to be similar in children and adults [32, 33], differences in respiratory droplet and aerosol formation [34], respiratory anatomy [35], or increased tropism of the virus in the upper vs. lower airways in children [36, 37] could contribute to the suggested differences in virus transmission. It should be noted that transmission dynamics may be different for more recent variants of SARS-CoV-2. For the B.1.617. (delta) variant, viral loads appeared to be higher than for the B.1.1.7 (alpha) variant [38], and significantly increased with age [39]. Research on the delta variant [40] and preliminary reports on the omicron variant [41, 42] indicate their increased airway replication fitness compared to the ancestral and alpha variants, and this could also significantly influence the infectiousness of children with COVID-19.

In our cohort, we provide a longitudinal follow-up of SARS-CoV-2 serology over 9 months after symptomatic illness, showing that children as well as adults retain both anti-spike IgG and anti-nucleocapsid IgA/IgG/IgM antibodies up to 270 days post-symptom onset (PSO). Despite the time elapsed since the beginning of the pandemic, long-term follow-up of quantitative serum antibody measurements such as ours for the ancestral SARS-CoV-2 are scarce in adults and even less has been reported for children. A follow-up of antibodies against SARS-CoV-2 in people living in Wuhan [17], as well as in Jiangsu [43], China, showed antibodies to persist in a majority of cases over six months. However, the authors did not provide data according to time since symptom onset or the age of the participants. Previously, persistence of antibodies in children based on two serological tests 62 days apart was reported, but time since symptom onset was also unknown [44]. One study in a somewhat comparable pediatric cohort showed also detectable IgG levels after 6 months and also in a single child 9 months after infection [45]; however, the cohort did not include adult contacts for comparison. A detailed immunological follow-up of adults [46] after SARS-CoV-2 infection showed similar results 6 to 8 months PSO. Compared to adult family members, children in our cohort showed on average higher anti-spike IgG antibody levels in early reconvalescence (less than 121 days PSO). A recent work [47] including children aged 3–11 years similarly showed higher antibody levels in children compared to adults against multiple epitopes of SARS-CoV-2, as well as persistent anti-spike antibodies in 14 out of 16 children tested at least 12 months after seroconversion. These findings as well as ours are in contrast to some earlier reports showing lower neutralizing activity [48] or similar anti-spike IgG levels [49] compared to adults, albeit addressed in older pediatric subcohorts. Pre-existing memory B cells and natural antibodies [50] or T cell memory [51, 52] in children on account of more frequent common-cold coronavirus infections may lead to quicker and more efficient SARS-CoV-2-specific immune response and thus higher initial antibody concentrations. The same mechanisms might also contribute to the milder clinical course of SARS-CoV-2 that is characteristic in children. Moreover, as demonstrated in our cohort, anti-spike IgG levels remained higher in children than adults over 193 days PSO, even though a more rapid decline in serum antibody concentrations of children was observed initially.

There are limitations to this study. Teenagers were relatively underrepresented when compared to younger children and Hamburg census data. The retrospective nature of serological testing in this study did not allow for confirmation of infection by direct virus detection (PCR); therefore, seropositivity is used as a surrogate marker for past infection. Furthermore, PCR test results from study participants from the time of infection were not available and index cases in family clusters were identified based on patient history of symptomatic illness (Supplementary Table S2). A selection bias needs to be considered when interpreting our findings. First, our study included over 50% volunteers; second, blood draws were more likely to have been carried out in older children (age distribution of children with available serology compared to the whole study population in Supplementary Figure S3). In this study, no serum neutralization tests have been performed. Instead, seropositivity was defined based on two different serum antibody tests, both of which have shown good correlation with plaque reduction neutralization tests [53, 54]. While this approach does not measure direct antibody-mediated protective immunity, it reduces the probability of false positive tests, especially when multiple family members are assessed. Since the samples used for antibody measurements in this study were obtained after the first 2020 wave of infections, they represent infections with the ancestral SARS-CoV-2 which has now been replaced by more recent viral variants. In spite of this, our data is relevant to antibody responses after vaccination, because all current vaccines are based on inactivated virus or sequences from the ancestral SARS-CoV-2 [55–58]. The setting in which our data was obtained is unique because widespread vaccination and possible repeat infections would complicate designing similar prospective studies in the present day.

In this work, we demonstrated a less than 100% seroprevalence within families in case of infection with the SARS-CoV-2 ancestral variant. Furthermore, seroprevalence within households was lower in case of pediatric than adult index cases. Our findings showing a sustained long-term antibody response in the pediatric population after the initial infection wave of SARS-CoV-2 is relevant for vaccination efforts targeting children as well as newly emerging viral variants.

## Supplementary Information

Below is the link to the electronic supplementary material.

Supplementary file1 (DOCX 11584 KB)

## Data Availability

The datasets generated during and/or analyzed during the current study are available from the corresponding author on reasonable request.
