# Supplementary material for: Long-Term Antibody Response to SARS-CoV-2 in Children
Source: J Clin Immunol. 2022 Sep 19;43(1):46–56. doi: 10.1007/s10875-022-01355-w (PMC9483535; doi:10.1007/s10875-022-01355-w)

**Supplementary Appendix to manuscript entitled**

**Long‑term antibody response to SARS-CoV-2 in children**

Table of Contents

[Supplementary Methods 2](#_Toc109664048)

[Recruitment process and exclusion 2](#_Toc109664049)

[Clinical samples 2](#_Toc109664050)

[Viral RNA extraction and qPCR 2](#_Toc109664051)

[Serology tests 3](#_Toc109664052)

[Supplementary Tables and Figures 4](#_Toc109664053)

[Table S1. Underlying medical conditions in C19.CHILD study population 4](#_Toc109664054)

[Table S2. Overview of the 45 families recalled for the follow-up phase. 5](#_Toc109664055)

[Figure S1. Seroprevalence distribution across age in children under 18 years of age. 11](#_Toc109664056)

[Figure S2. Cumulative incidence per 100,000 in the general population of the city of Hamburg 12](#_Toc109664057)

[Figure S3. Age distribution of the C19.CHILD cohort with available serology 12](#_Toc109664058)

# **Supplementary Methods**

##
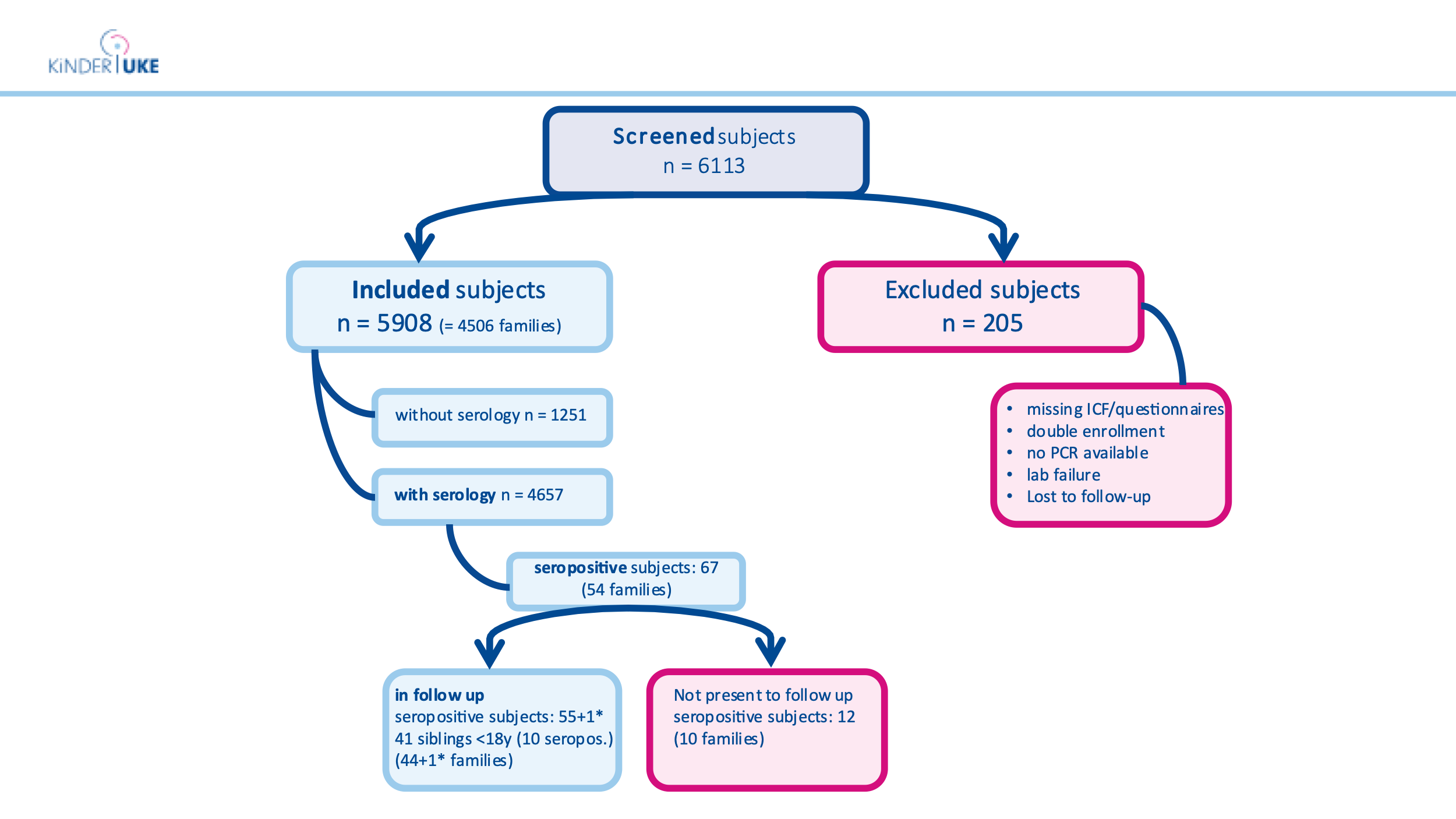
**Recruitment process and exclusion**

*subject had a discordant screening result but presented with family at follow up and had a seropositive result

## **Clinical samples**

Nasopharyngeal swabs were collected and preserved in 3 ml of universal transport medium (UTM; Miraclean Technology). All swab samples were inactivated and used for viral RNA isolation within 72 hours. Blood samples were processed within 24 hours for serum separation.

## **Viral RNA extraction and qPCR**

RNA was extracted from clinical samples using the NucleoSpin 96 Virus Core Kit (Macherey-Nagel). Samples were inactivated upon addition of the kit lysis buffer (RAV-1) in 1:4 ratio (v/v) and incubated for 30 min before RNA isolation. The kit manufacturer´s instructions were adapted for automated RNA extraction using a Tecan Freedom Evo liquid handler system equipped with a TeVacs vacuum unit (Tecan).

E- and S-gene quantitative PCR was used for qualitative detection of lineage B-betacoronavirus (B-βCoV) and SARS-CoV-2. RNA reverse transcription and PCR were performed using RealStar SARS-CoV-2 RT-PCR Kit 1.0 (altona Diagnostics). An RNA internal control (RealStar kit component) was added to the inactivated samples prior to RNA isolation for control of RNA isolation and RT-PCR inhibition. Multiplex qPCR using distinct probe dyes for the viral E-gene (B-βCoV specific), the S-gene (SARS-CoV-2 specific), and the internal control, allowed for parallel detection of the different targets. The RT-PCR reaction comprised a 30 µL reaction containing 10 µL of extracted RNA and 20 µL of RT-PCR reaction mix. Thermal cycling consisted of an incubation at 55 °C for 10 min for reverse transcription, followed by 95 °C for 2 min and then 45 cycles of 95 °C for 15 sec, 55 °C for 45 sec and 72 °C for 15 sec. A QuantStudio 12K Flex Real-Time PCR System equipped with a standard 96-well block (Applied Biosystems) or a CFX96 Connect cycler (Bio-Rad Laboratories) were used. *In vitro* transcribed E-gene RNA was spiked into RAV-1 before RNA isolation and used as a positive control, while the negative control consisted of UTM in RAV-1. In addition, an E- and S-gene positive control provided by the RealStar kit was used in the RT-PCR reaction.

The assessment of the limit of detection (LOD) of the test was done using 10 replicates of a dilution series of EDX SARS-CoV-2 synthetic RNA (Exact Diagnostics) containing five gene targets (E, N, ORF1ab, RdRP and S Genes of SARS-CoV-2) in UTM and RAV-1. The LOD was 156 copies/mL for SARS-CoV-2 specific S-gene, and 313 copies/mL for B-ßCoV E-gene, with a detection rate over 95 %.

For RT-PCR data analysis, QuantStudio 12K Flex (Applied Biosystems) or CFX Manager (Bio-Rad Laboratories) software were used. Samples were considered valid if the Ct value for the internal control was lower than 35. Amplification of S-/E-gene, independent of the Ct, was considered positive.

## **Serology tests**

After serum separation, samples were directly used for antibody testing or stored at −20 °C prior to analysis.

Sample screening was performed using the Elecsys® AntiSARS-CoV-2 assay (Roche) in a cobas e411 system and a cut-off index above 1 was used to define positivity, following the manufacturer’s instructions. Elecsys® AntiSARS-CoV-2 detects antibodies (IgA, IgM, IgG) against the SARS-CoV-2 nucleocapsid protein with a 99.8 % specificity (according to Roche).

The LIAISON® SARS-CoV-2 S1/S2 IgG assay (DiaSorin) was used as a confirmatory assay. LIAISON® assay was performed using a Liaison XL system, following the manufacturers’ recommendations. 10 AU/mL was used as the cut-off for positivity. Assay specificity is 98.5 %, according to the manufacturer, for detection of IgG antibodies against the spike S1 and S2 proteins.

# **Supplementary Tables and Figures**

**Table S1. Underlying medical conditions in C19.CHILD study population**

Distribution of participants with underlying medical conditions in the population is presented as counts (percent). Data is compared using Fisher’s exact test.

|  | **Negative in either test (N=4590)** | **Positive Roche & DiaSorin (N=67)** | **Total  (N=4657)** | **P value** |
| --- | --- | --- | --- | --- |
| Underlying condition | 1334 (29.1 % %) | 16 (23.9 %) | 1350 (29.0 %) | 0.42 |
| thereof |  |  |  |  |
| Respiratory | 157 (11.8 %) | 2 (12.5 %) | 159 (11.8 %) | >0.99 |
| Hepatic | 42 (3.1 %) | 0 (0.0 %) | 42 (3.1 %) | >0.99 |
| Onco-/Hematologic | 220 (16.5 %) | 1 (6.3 %) | 221 (16.4 %) | 0.38 |
| Immunologic | 91 (6.8 %) | 2 (12.5 %) | 93 (6.9 %) | 0.39 |
| Neurologic | 163 (12.2 %) | 1 (6.3 %) | 164 (12.1 %) | 0.73 |
| Rheumatologic | 63 (4.7 %) | 1 (6.3 %) | 64 (4.7 %) | 0.61 |
| Cardiovascular | 216 (16.2 %) | 3 (18.8 %) | 219 (16.2 %) | >0.99 |
| Metabolic | 122 (9.1 %) | 1 (6.3 %) | 123 (9.1 %) | >0.99 |
| Renal | 164 (12.3 %) | 1 (6.3 %) | 165 (12.2 %) | 0.73 |
| Inflammatory Bowel Disease | 53 (4.0 %) | 1 (6.3 %) | 54 (4.0 %) | 0.54 |
| Endocrine | 32 (2.4 %) | 0 (0.0 %) | 32 (2.4 %) | >0.99 |
| Atopic | 106 (7.9 %) | 3 (18.8 %) | 109 (8.1 %) | 0.21 |
| Liver Transplantation | 21 (1.6 %) | 0 (0.0 %) | 21 (1.6 %) | >0.99 |
| Kidney Transplantation | 24 (1.8 %) | 0 (0.0 %) | 24 (1.8 %) | >0.99 |
| Transplantation | 80 (6.0 %) | 0 (0.0 %) | 80 (5.9 %) | 0.63 |
| Hematopoietic Stem Cell Transplantation | 24 (1.8 %) | 0 (0.0 %) | 24 (1.8 %) | >0.99 |
| Solid Organ Transplantation | 46 (3.4 %) | 0 (0.0 %) | 46 (3.4 %) | >0.99 |
| Psychologic | 18 (1.3 %) | 0 (0.0 %) | 18 (1.3 %) | >0.99 |
| Trisomy 21 | 11 (0.8 %) | 0 (0.0 %) | 11 (0.8 %) | >0.99 |

## **Table S2. Overview of the 45 families recalled for the follow-up phase.**

Seven children refused repeated blood draw in the follow-up phase. Here the result from the screening phase was used for the calculation of the seroprevalence within families (participants are marked with*). For three families one family member refused blood draw (Family IDs: 28,29,73) and no serology data is available. The table is also available as a separate Excel file in the online supplementary material.

| **Family ID** | **DiaSorin** | **Roche** | **Age** | **Relationship** | **Self-reported symptoms** | **Time of symptomatic illness** | **Index case** |
| --- | --- | --- | --- | --- | --- | --- | --- |
|  | **IgG** | **IgM** | **(y)** |  |  |  |  |
| 3 | positive | positive | 2 | screening positive | yes |  | parent |
| 3* | negative | negative | 6 | sibling | no |  |  |
| 3 | positive | positive | 38 | parent | NA |  |  |
| 3 | positive | positive | 38 | parent | yes | March 2020 |  |
| 4 | positive | positive | 17 | screening positive | yes | March 2020 | screening positive |
| 4 | negative | negative | 14 | sibling | no |  |  |
| 4 | negative | negative | 12 | sibling | no |  |  |
| 4 | negative | negative | 57 | parent | no |  |  |
| 4 | negative | negative | 19 | sibling | no |  |  |
| 4 | negative | negative | 52 | parent | no |  |  |
| 8 | positive | positive | 6 | screening positive | no |  | parent |
| 8 | positive | positive | 10 | screening positive | no |  |  |
| 8 | positive | positive | 44 | parent | yes |  |  |
| 8 | negative | negative | 78 | grandparent | yes |  |  |
| 8 | positive | positive | 42 | parent | yes |  |  |
| 16 | positive | positive | 1 | screening positive | yes | March 2020 | parent |
| 16 | negative | negative | 3 | sibling | yes |  |  |
| 16 | positive | positive | 34 | parent | yes | March 2020 |  |
| 16 | positive | positive | 33 | parent | yes | March 2020 |  |
| 17 | positive | positive | 15 | screening positive | no |  | sibling (>18y) |
| 17 | positive | positive | 55 | parent | no |  |  |
| 17 | positive | positive | 49 | parent | no |  |  |
| 17 | positive | positive | 23 | sibling | no |  |  |
| 17 | positive | positive | 19 | sibling | yes | March 2020 |  |
| 18 | positive | positive | 7 | screening positive | yes | March 2020 | parent |
| 18 | negative | positive | 3 | screening positive | no |  |  |
| 18 | negative | negative | 9 | sibling | no |  |  |
| 18 | positive | positive | 49 | parent | yes | March 2020 |  |
| 18 | positive | positive | 39 | parent | yes | March 2020 |  |
| 19 | positive | positive | 6 | screening positive | no |  | parent |
| 19 | positive | positive | 11 | screening positive | no |  |  |
| 19 | positive | positive | 47 | parent | yes |  |  |
| 19 | negative | negative | 46 | parent | yes | March 2020 |  |
| 20 | positive | positive | 15 | screening positive | no |  | NA |
| 20 | negative | negative | 63 | parent | no |  |  |
| 20 | negative | negative | 56 | parent | no |  |  |
| 23 | positive | positive | 13 | screening positive | yes | March 2020 | NA |
| 23 | negative | negative | 4 | sibling | yes |  |  |
| 23 | negative | negative | 1 | sibling | yes |  |  |
| 23 | negative | negative | 9 | sibling | yes |  |  |
| 23 | negative | negative | 39 | parent | yes |  |  |
| 23 | negative | negative | 35 | parent | yes |  |  |
| 26 | positive | positive | 7 | screening positive | no |  | parent |
| 26 | negative | negative | 3 | sibling | no |  |  |
| 26 | positive | positive | 3 | sibling | no |  |  |
| 26 | negative | negative | 47 | parent | no |  |  |
| 26 | positive | positive | 53 | parent | yes | March 2020 |  |
| 26 | positive | positive | 24 | other | no |  |  |
| 27 | positive | positive | 0 | screening positive | no |  | parent |
| 27 | positive | positive | 6 | sibling | no |  |  |
| 27 | positive | positive | 35 | parent | yes | March 2020 |  |
| 27 | positive | positive | 32 | parent | yes | March 2020 |  |
| 28* | positive | positive | 11 | screening positive | yes |  | NA |
| 28 | negative | negative | 5 | sibling | no |  |  |
| 28 |  |  | 8 | sibling | no |  |  |
| 28 | negative | negative | 8 | sibling | yes |  |  |
| 28 | negative | negative | 41 | parent | yes |  |  |
| 28 | positive | positive | 44 | parent | yes |  |  |
| 29 | positive | positive | 10 | screening positive | no |  | parent |
| 29 |  |  | 3 | sibling | no |  |  |
| 29 | negative | negative | 7 | sibling | no |  |  |
| 29 | positive | positive | 42 | parent | yes | Apr 20 |  |
| 29 | positive | positive | 45 | parent | yes | Apr 20 |  |
| 30 | positive | positive | 12 | screening positive | no |  | sibling |
| 30 | negative | negative | 11 | sibling | yes | March 2020 |  |
| 30 | positive | positive | 44 | parent | yes | March 2020 |  |
| 30 | negative | positive | 45 | parent | NA |  |  |
| 32* | positive | positive | 6 | screening positive | no |  | NA |
| 32 | positive | positive | 25 | parent | yes |  |  |
| 33 | positive | positive | 16 | screening positive | yes | Apr 20 | parent |
| 33 | positive | positive | 11 | screening positive | yes | Apr 20 |  |
| 33 | positive | positive | 14 | screening positive | yes |  |  |
| 33 | positive | positive | 50 | parent | yes | Apr 20 |  |
| 33 | negative | positive | 50 | parent | yes | March 2020 |  |
| 34 | positive | positive | 11 | screening positive | no |  | screening positive |
| 34 | positive | positive | 48 | parent | no |  |  |
| 34 | negative | negative | 46 | parent | no |  |  |
| 35 | positive | positive | 3 | screening positive | yes | March 2020 | screening positive |
| 35 | positive | positive | 39 | parent | yes | March 2020 |  |
| 36 | positive | positive | 17 | screening positive | yes | March 2020 | screening positive |
| 36 | negative | negative | 12 | sibling | no |  |  |
| 36 | negative | negative | 50 | parent | no |  |  |
| 36 | negative | negative | 51 | parent | no |  |  |
| 37 | positive | positive | 10 | screening positive | yes | March 2020 | screening positive |
| 37 | positive | negative | 3 | sibling | yes | March 2020 |  |
| 37 | negative | negative | 4 | sibling | yes | March 2020 |  |
| 37 | negative | negative | 29 | other | yes | March 2020 |  |
| 37 | negative | negative | 55 | parent | yes | March 2020 |  |
| 37 | negative | negative | 30 | sibling | yes | March 2020 |  |
| 37 | negative | negative | 76 | grandparent | no |  |  |
| 37 | negative | negative | 53 | parent | no |  |  |
| 37 | negative | negative | 19 | sibling | yes | March 2020 |  |
| 39 | positive | positive | 15 | screening positive | yes |  | NA |
| 39 | negative | negative | 12 | sibling | no |  |  |
| 39 | positive | positive | 9 | sibling | no |  |  |
| 39 | negative | negative | 44 | parent | yes |  |  |
| 39 | negative | negative | 45 | parent | yes |  |  |
| 40 | positive | positive | 7 | screening positive | yes |  | parent |
| 40 | positive | positive | 14 | screening positive | yes | March 2020 |  |
| 40 | negative | negative | 11 | sibling | yes |  |  |
| 40 | positive | positive | 44 | parent | yes | March 2020 |  |
| 40 | positive | positive | 44 | parent | yes | March 2020 |  |
| 41 | positive | positive | 11 | screening positive | no |  | parent |
| 41 | positive | positive | 20 | sibling | yes | March 2020 |  |
| 41 | negative | negative | 23 | sibling | yes | March 2020 |  |
| 41 | positive | positive | 53 | parent | yes | March 2020 |  |
| 41 | positive | positive | 51 | parent | yes |  |  |
| 45 | positive | positive | 9 | screening positive | no |  | NA |
| 45 | negative | positive | 13 | screening positive | no |  |  |
| 45 | negative | negative | 53 | parent | no |  |  |
| 45 | negative | negative | 47 | parent | no |  |  |
| 54 | positive | positive | 13 | screening positive | no |  | screening positive |
| 54* | positive | positive | 13 | screening positive | yes |  |  |
| 54 | positive | positive | 16 | screening positive | yes | March 2020 |  |
| 54 | positive | positive | 49 | parent | yes | March 2020 |  |
| 54 | positive | positive | 52 | parent | no |  |  |
| 55 | positive | positive | 6 | screening positive | yes | Apr 20 | parent |
| 55 | negative | negative | 44 | parent | yes | March 2020 |  |
| 55 | positive | positive | 41 | parent | no |  |  |
| 56 | positive | positive | 12 | screening positive | no |  | parent |
| 56 | positive | positive | 5 | sibling | yes | March 2020 |  |
| 56 | positive | positive | 47 | parent | yes | March 2020 |  |
| 56 | positive | positive | 46 | parent | yes | March 2020 |  |
| 57 | positive | positive | 15 | screening positive | no |  | parent |
| 57 | positive | positive | 17 | sibling | no |  |  |
| 57 | positive | positive | 10 | sibling | no |  |  |
| 57 | positive | positive | 3 | sibling | no |  |  |
| 57 | positive | positive | 52 | parent | yes | May 2020 |  |
| 57 | positive | positive | 43 | parent | yes | May 2020 |  |
| 66 | positive | positive | 17 | screening positive | yes | March 2020 | sibling |
| 66 | negative | negative | 12 | sibling | yes | March 2020 |  |
| 66 | negative | negative | 12 | sibling | no |  |  |
| 66 | negative | negative | 55 | other | yes | March 2020 |  |
| 66 | negative | negative | 48 | parent | no |  |  |
| 66 | negative | negative | 54 | parent | yes | March 2020 |  |
| 68 | positive | positive | 9 | screening positive | no |  | parent |
| 68 | negative | negative | 7 | sibling | no |  |  |
| 68 | positive | positive | 40 | parent | yes | March 2020 |  |
| 68 | positive | positive | 41 | parent | yes | March 2020 |  |
| 69 | positive | positive | 17 | screening positive | yes | March 2020 | NA |
| 69 | positive | positive | 54 | parent | yes | March 2020 |  |
| 70* | positive | positive | 4 | screening positive | yes | March 2020 | parent |
| 70* | negative | negative | 5 | sibling | no |  |  |
| 70 | positive | positive | 45 | parent | yes | March 2020 |  |
| 70 | positive | positive | 45 | parent | yes | March 2020 |  |
| 72 | positive | positive | 13 | screening positive | no |  | parent |
| 72 | negative | negative | 49 | parent | yes | March 2020 |  |
| 73 | positive | positive | 15 | screening positive | yes | March 2020 | screening positive |
| 73 | positive | positive | 41 | parent | yes | March 2020 |  |
| 73 |  |  | 48 | parent | yes | March 2020 |  |
| 76 | positive | positive | 13 | screening positive | yes | March 2020 | parent |
| 76 | positive | positive | 9 | screening positive | yes |  |  |
| 76 | negative | negative | 16 | sibling | yes |  |  |
| 76 | positive | positive | 18 | sibling | yes |  |  |
| 76 | positive | positive | 49 | parent | yes | March 2020 |  |
| 76 | negative | positive | 47 | parent | yes |  |  |
| 84 | positive | positive | 12 | screening positive | yes |  | parent |
| 84 | negative | positive | 9 | screening positive | no |  |  |
| 84 | positive | positive | 49 | parent | yes |  |  |
| 84 | positive | positive | 48 | parent | yes | March 2020 |  |
| 89 | positive | positive | 9 | screening positive | yes | March 2020 | screening positive |
| 89 | negative | negative | 6 | sibling | yes |  |  |
| 89 | negative | negative | 43 | parent | yes |  |  |
| 89 | negative | negative | 42 | parent | yes |  |  |
| 107 | positive | positive | 16 | screening positive | yes | March 2020 | screening positive |
| 107 | negative | negative | 6 | sibling | no |  |  |
| 107 | positive | negative | 8 | sibling | no |  |  |
| 107 | positive | positive | 46 | parent | yes | March 2020 |  |
| 107 | positive | positive | 46 | parent | yes | March 2020 |  |
| 108 | positive | positive | 10 | screening positive | yes | March 2020 | parent |
| 108 | negative | negative | 13 | sibling | no |  |  |
| 108 | negative | negative | 10 | sibling | no |  |  |
| 108 | positive | positive | 47 | parent | yes | March 2020 |  |
| 108 | negative | positive | 48 | parent | yes | March 2020 |  |
| 109 | positive | positive | 10 | screening positive | yes | March 2020 | parent |
| 109 | negative | negative | 50 | parent | NA |  |  |
| 109 | positive | positive | 46 | parent | yes | March 2020 |  |
| 110 | positive | positive | 14 | screening positive | yes | March 2020 | parent |
| 110 | positive | positive | 16 | sibling | yes |  |  |
| 110 | positive | positive | 4 | sibling | yes |  |  |
| 110 | positive | positive | 38 | parent | yes | March 2020 |  |
| 110 | positive | positive | 35 | parent | yes | March 2020 |  |
| 111 | positive | positive | 10 | screening positive | no |  | parent |
| 111 | negative | negative | 12 | sibling | no |  |  |
| 111 | negative | negative | 74 | grandparent | no |  |  |
| 111 | negative | positive | 51 | parent | yes |  |  |
| 111 | negative | negative | 78 | grandparent | no |  |  |
| 111 | negative | positive | 48 | parent | yes | March 2020 |  |
| 112 | positive | positive | 9 | screening positive | no |  | parent |
| 112* | positive | positive | 6 | screening positive | yes | March 2020 |  |
| 112 | negative | negative | 42 | parent | no |  |  |
| 112 | positive | positive | 43 | parent | yes | March 2020 |  |
| 113 | positive | positive | 4 | screening positive | yes |  | parent |
| 113 | positive | positive | 3 | sibling | no |  |  |
| 113 | positive | positive | 38 | parent | yes | March 2020 |  |
| 113 | negative | positive | 34 | parent | yes | March 2020 |  |
| 114 | positive | positive | 16 | screening positive | yes | March 2020 | parent |
| 114 | positive | positive | 13 | screening positive | yes | March 2020 |  |
| 114 | positive | positive | 11 | screening positive | no |  |  |
| 114 | negative | positive | 52 | parent | yes | March 2020 |  |
| 114 | positive | positive | 47 | parent | yes | March 2020 |  |

NA: not available

## **Figure S1. Seroprevalence distribution across age in children under 18 years of age.**

The black dots indicate the seroprevalence per age. The calculated logistic regression model and corresponding 95% CI are shown (blue line and grey area). The seroprevalence increases with an odds ratio of 1.11 (95 % CI: 1.03-1.21, P=0.009) per year of age. For the 3 different age groups (0 - <6, 6 - <12 and 12 - <18 years), the mean seroprevalence and respective 95% CI are depicted above the logistic regression model. Included are 4657 children that participated in the screening phase, including serologic testing. Seropositivity is defined based on a positive result for both SARS-CoV-2 anti-spike IgG and anti-nucleocapsid IgA/IgG/IgM.

**
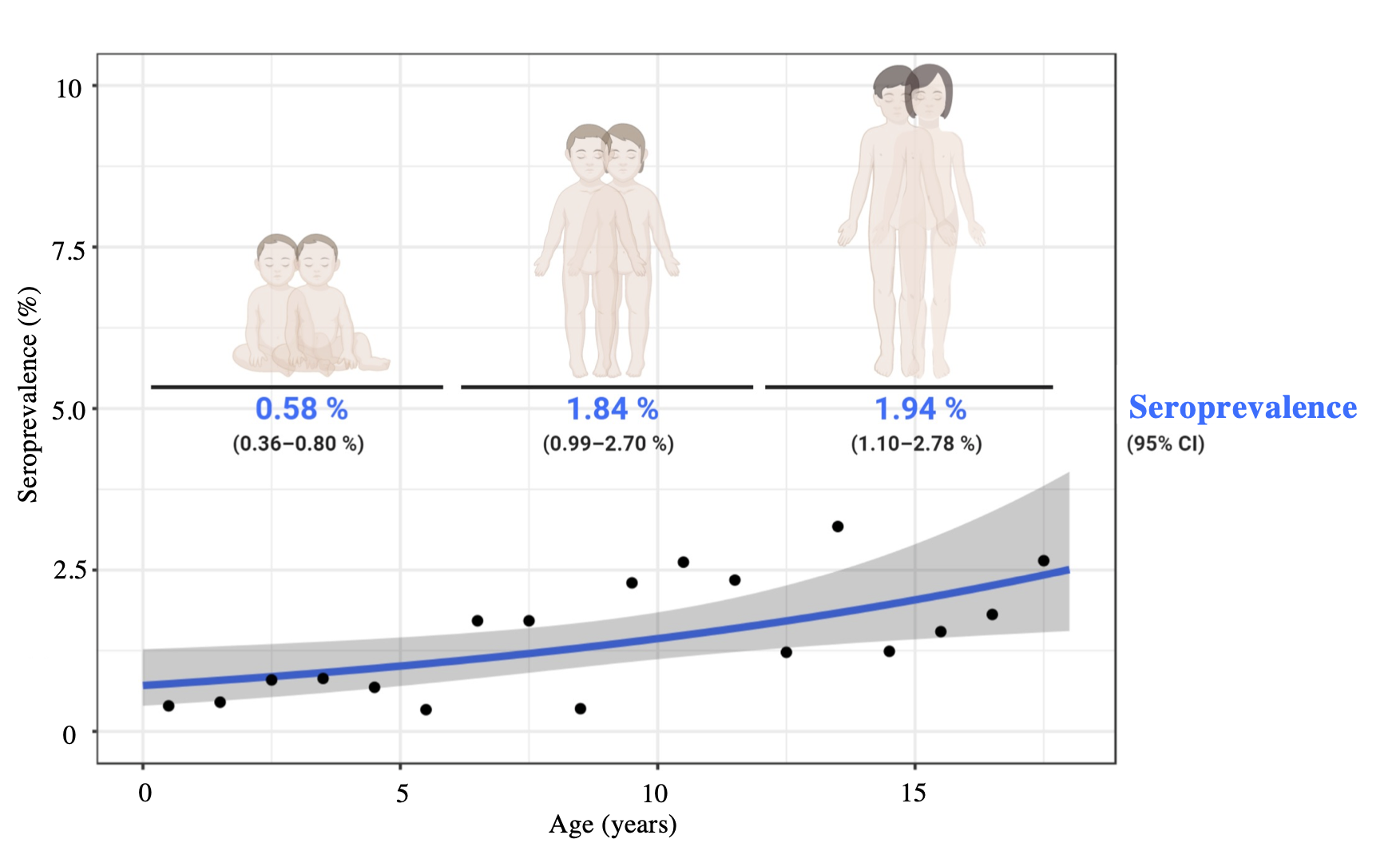
**

## **Figure S2. Cumulative incidence per 100,000 in the general population of the city of Hamburg**

Data taken from daily situation reports of the Robert Koch Institute (https://www.rki.de/DE/Content/InfAZ/N/Neuartiges_Coronavirus/Situationsberichte/Gesamt.html), before, during, and after the screening phase of the C19.CHILD study.

## **Figure S3. Age distribution of the C19.CHILD cohort with available serology**

Comparison of the age distribution of the C19.CHILD cohort with available serology (light blue) and the general C19.CHILD cohort (light grey).


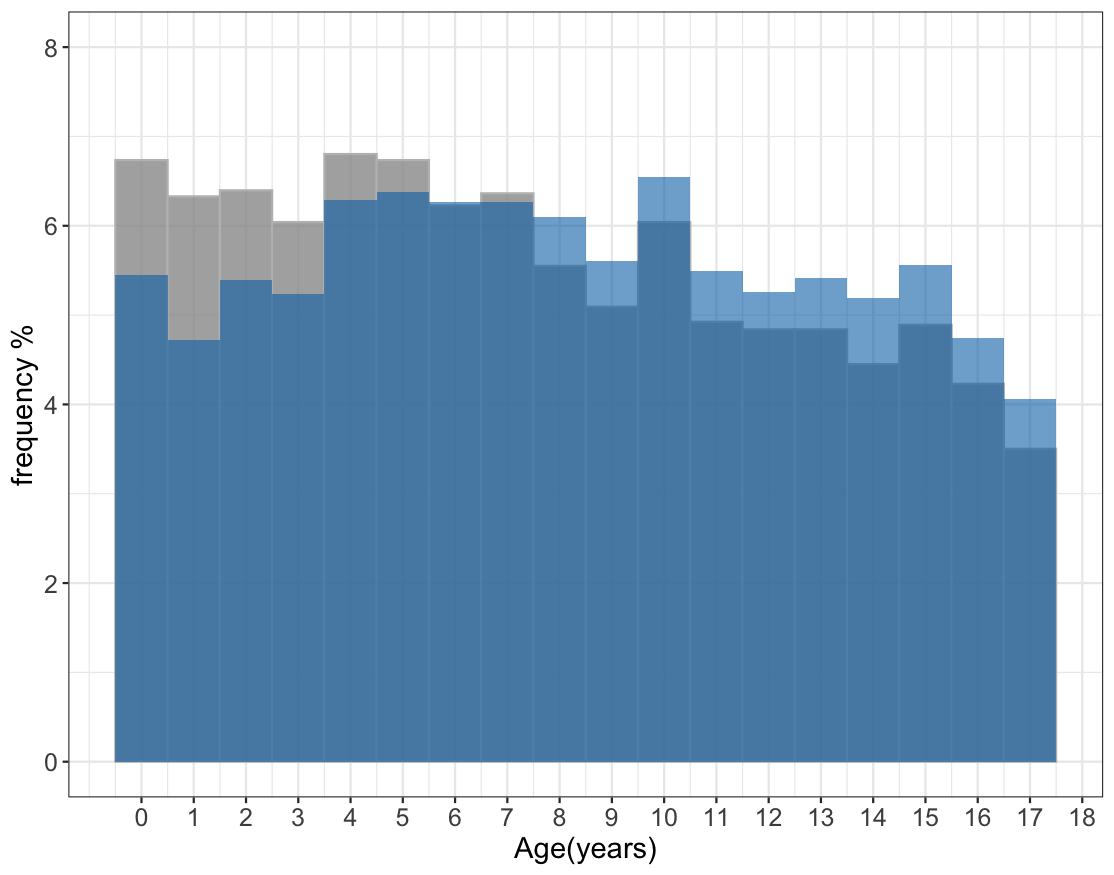

Supplement: Supplementary file 1 — Supplementary file1 (DOCX 11584 KB) [file 10875_2022_1355_MOESM1_ESM.docx]
